# Sublethal Acetamiprid Exposure Induces Ovarian Degeneration and HSP70/HSP90 Overexpression in Honey Bee Queens (*Apis mellifera*)

**DOI:** 10.3390/vetsci13090876

**Published:** 2026-08-27

**Authors:** Yasmine Ait Kara, Karima Benfodil, Assia Cherifi, Ourdia Sadeddine Zennouche, Chahra Dahmani, Chafika Mouhoub Sayah, Giorgia Rosato, Rebecca Leandri, Karen Power

**Affiliations:** 1 Laboratorie de Gestion et Valorisation des Ressources Naturelles et Assurance Qualite, Faculté SNVST, Université de Bouira, Bouira 10000, Algeria; y.aitkara@univ-bouira.dz (Y.A.K.); c.dahmani@univ-bouira.dz (C.D.); ch.mouhoub@univ-bouira.dz (C.M.S.); 2 Laboratory of Biotechnology and Protection of Agricoltural and Natural Ecosystems, Faculty of Nature and Life Sciences and Earth Sciences, Bouira University, Bouira 10000, Algeria; k.benfodil@univ-bouira.dz; 3 Faculté des Sciences de la Nature et de la Vie et des Sciences de la Terre, Université de Bouira, Bouira 10000, Algeria; a.cherifi@univ-bouira.dz; 4 Associated Laboratory in Marine Ecosystem and Aquacole, Faculty of Natural and Life Sciences, Université de Bejaia, Bejaia 06000, Algeria; ourdia.zennouche@univ-bejaia.dz; 5 Department of Biology, University of Naples ‘Federico II’, Via Vicinale Cupa Cinthia 21, 80216 Napoli, Italy; giorgia.rosato@unina.it (G.R.); rebecca.leandri@unina.it (R.L.)

**Keywords:** histopathology, immunohistochemistry, insecticides, ovaries, pollinators, reproductive health

## Abstract

The health of the honey bee (*Apis mellifera*) is threatened by a variety of biotic and abiotic stressors, among which pesticide exposure remains a major factor. Acetamiprid is a neonicotinoid still authorized in the European Union despite the known toxic effects exerted on honey bees. To date, its impact on queens’ health remains largely unexplored although colony survival is strongly dependent on the reproductive success of the queen. In this study, we evaluated the effects of a 7-day sublethal exposure to acetamiprid (20 μg/L) on the ovarian structure using histological and immunohistochemical approaches. The histological analysis revealed structural alterations in the ovaries of exposed queens, including disorganization of the germarium and vitellarium, cytoplasmatic vacuolization in the interstitial connective tissue, oocyte degeneration and pyknotic nuclei in nurse cells, indicative of apoptosis. In addition, immunohistochemical analysis revealed increased expression of HSP70 and HSP90, indicating a disruption of cellular proteostasis and cellular stress. These findings suggest that sublethal short-term exposure to acetamiprid may directly affect the queen bee reproductive apparatus.

## Simple Summary

Queen bees are essential to the survival of the *Apis mellifera* colony, as they are responsible for laying eggs and maintaining the bee population. For this reason, their reproductive health is crucial for the stability of the entire hive and is therefore an object of growing scientific interest. In this study, we evaluated the effects of the pesticide acetamiprid, currently approved in the European Union, on the queen bee reproductive system. Short-term exposure to low concentrations induced histological alterations in the ovary and increased the expression of stress-related proteins (HSP70 and HSP90). These findings suggest that sublethal short-term exposure to acetamiprid may directly affect the queen bee reproductive apparatus.

## 1. Introduction

The honey bee (*Apis mellifera*) is considered the most widespread pollinator species [1] due to its economic importance and crucial role in sustaining both natural and agricultural biodiversity through the pollination of flowering plants and economically important crops [2,3,4,5]. This service is vital to high-value crops, as their yield and quality can be significantly influenced by bee activity [6,7]. Beyond pollination, the honey bee contributes directly to the economy through the production of honey, pollen, wax, royal jelly and propolis, which are used in nutrition and medicine [8,9]. In addition, honey bees are widely recognized as effective bioindicators of environmental pollution, providing valuable insights into the effects of anthropogenic activities and supporting the development of strategies aimed at protecting both ecosystems and human health [10].

Honey bee colonies depend on the reproductive capacity of a single queen to sustain their population [11]. The honey bee queen possesses a pair of highly developed ovaries, each containing 150 to 180 ovarioles, and following a “one-time” polyandrous mating, spermatozoa are stored in a spermatheca, ready to fertilize the eggs [12]. A queen can lay up to 1500 eggs per day, therefore any decline in her reproductive capacity poses a direct and immediate threat to colony survival and stability [13]. Indeed, the loss of the queen has been recognized as one of the most significant factors contributing to colony losses, particularly outside of the typical queen rearing season [14,15].

Honey bees are exposed to several factors that could affect their survival and health, including pathogens, parasites, climate changes, invasive species, malnutrition and pesticide exposure [16,17]. In particular, exposure to xenobiotic compounds occurs through multiple pathways, including the ingestion of contaminated nectar, pollen, and water, as well as through direct contact with treated surfaces, leading to the accumulation of residues on their bodies [18,19,20,21]. Honey bees are particularly susceptible to pesticides, as their genome contains fewer genes that encode xenobiotic detoxifying enzymes compared to other insects [22]. In particular, the global increase in the application of neonicotinoid pesticides over the past decade has been considered a key factor in the decline of honey bee colonies [23]. Since their introduction in the early 1990s, neonicotinoids have rapidly become the most widely used class of insecticides worldwide. Their extensive use has raised growing concerns about their environmental impacts, particularly on pollinators [24]. Indeed, neonicotinoids are considered among the pesticides that are most toxic to bees [25]. Due to their slow degradation, following application, neonicotinoids may persist in soil and treated plants for months or even years [26,27]. Consequently, neonicotinoid residues can accumulate in nectar and pollen [24,28], be ingested by honey bees, and act as neurotoxins that interfere with the insect nervous system by binding to acetylcholine receptors [29,30].

While imidacloprid, clothianidin, and thiamethoxam have been banned or severely restricted for outdoor use on flowering crops in the European Union since 2018, acetamiprid remains the only neonicotinoid currently authorized for outdoor application in the EU, with its approval extended until 2033 (European Food Safety Authority (EFSA) 2016). In agroecosystems, it is commonly applied to control key insect pests belonging to the orders Hemiptera, Thysanoptera and Lepidoptera [31].

Most studies of pesticide toxicity have focused on honey bee workers [32,33,34], while the queen, despite her central role in colony survival and reproduction, has received comparatively little attention. Several studies have found that acetamiprid affects not only honey bee olfactory memory and cognitive performance [35,36], but also queen rearing [37]. Furthermore, while the ovarian toxicity of imidacloprid and thiamethoxam in queens has been investigated [38,39], no study has specifically addressed the effects of acetamiprid on queen reproductive physiology and ovarian structure. Several approaches have been employed to assess pesticide toxicity in honey bees, including biochemical, molecular, and behavioral analyses [40,41,42]. On the contrary, in honey bee ecopathological studies, histopathology and immunohistochemistry (IHC) have been applied to a lesser extent despite their ability to point out sublethal alterations. In particular, through the detection of stress-related proteins such as heat shock proteins (HSPs) [43,44], it is possible to identify response mechanisms to xenobiotic exposure. HSPs represent well recognized molecular biomarkers of cellular stress as they maintain cellular proteostasis, being involved in correct protein fold, refold of unstable proteins, and regulation of apoptosis [45].

This study aimed to investigate the impact of sublethal acetamiprid exposure on the ovaries of honey bee queens. Acetamiprid was selected as the study compound based on its current regulatory status and its prevalent agricultural use in the study region (Algeria). Histological analysis was performed to identify potential structural alterations in the ovarioles, while the expression levels of HSPs (HSP70 and HSP90) were analyzed through immunohistochemical techniques to evaluate the cellular stress response induced by pesticide exposure.

## 2. Materials and Methods

### 2.1. Animals and Experimental Design

The experiment was conducted at the University of Bouira, Algeria (36.3749° N latitude and 3.9020° E longitude), in July 2024. First histological assessment was performed in 2024, while additional histological sections and immunohistochemical analyses were performed in 2025 using the same paraffin-embedded ovarian samples. Honey bee queens (*Apis mellifera*) were intentionally raised for the experiment from healthy, well-maintained colonies. Colony health status was determined by standard apicultural criteria including colony strength, brood pattern, absence of disease symptoms, and controlled *Varroa destructor* infestation levels [46,47]. Selected queens were young (approximately 2 months old), naturally mated sister queens and showed no apparent morphological anomalies [48]. Under laboratory conditions, each queen was kept individually in an experimental cage (16 × 10 × 10 cm) with ten newly emerged (one-day old) workers originating from the same mother colony. This experimental design ensured a standardized social environment for all queens while minimizing potential variation among colonies. All cages were maintained under controlled conditions at 34.5 °C and 60% humidity, in darkness [49,50]. A 50% sucrose solution was supplied ad libitum throughout the experimental period [42]. After 5 days of acclimatation, queens were exposed to acetamiprid treatment.

### 2.2. Exposure Protocol

A total of 25 queens were subjected to a 7-day experimental exposure, a duration selected based on previous studies showing that sublethal effects of neonicotinoids on honey bee queens can be detected within the first week of exposure [13,51]. Individuals were randomly assigned to two experimental groups, each composed of a queen and ten worker honey bees: (a) control group (n = 10), fed a 50% sucrose solution (sucrose/water), and (b) treated group (n = 15), fed the same sucrose solution supplemented with 20 μg/L of acetamiprid. Administration of the solution to queens occurred indirectly via worker bee trophallaxis. The concentration of 20 μg/L had been identified as a possible environmentally relevant sublethal dose and used in colony exposure studies reporting significant physiological alterations in honey bee workers and queens [52,53]. The insecticide was administered using a commercial formulation (PICADOR 20% SL, 200 g/L active ingredient, Red Sun Co., Ltd., Nanjing, China). To maintain chemical stability and prevent microbial fermentation, feeding solutions were replaced every 24 h throughout the exposure period.

### 2.3. Dissection and Organ Removal

After the exposure period (day 7), the queens were anesthetized with chilling for 10 min at 4 °C. Dissection was carried out using a binocular magnifying glass with a 25× magnification via gentle traction of the terminal abdominal segments [54]. The ovaries were carefully isolated using micro instruments and then placed in tubes containing 10% buffered formalin for 24 h before histological analysis.

### 2.4. Histology

Ovarian samples were placed in embedding cassettes and routinely processed for histopathology, by graded ethanol dehydration, xylene clearing and paraffin embedding. Paraffin-embedded samples were shipped to the Histopathology and Diagnostics Core of the Department of Biology of the University of Naples Federico II. Then, 3 μm sections were obtained using a microtome (RM 2015, Leica Biosystems, Deer Park, IL, USA). Single sections were placed on the surface of hot water and then collected on slides and air-dried at room temperature for 12 h. Subsequently, the slides were stained with hematoxylin–eosin (H&E), as previously described [55], and finally mounted. Tissue preparations were examined by light microscopy (Axio Scope A1, Carl Zeiss S.p.A., Oberkochen, Germany), and images were acquired using a digital imaging system equipped with a microphotography camera (Axiocam 105 color, Carl Zeiss S.p.A., Oberkochen, Germany).

Histological evaluation of ovarian tissues was performed using a semi-quantitative scoring system based on established histopathological approaches [56,57]. The following parameters were assessed as indicators of tissue damage: follicular degeneration, cyto-plasmatic vacuolization, disorganization of ovarioles, pyknosis and nuclear condensation.

Five histological serial sections per queen were evaluated, and all visible ovarioles within the selected sections were examined. For each queen, a score ranging from 0 to 3 was assigned according to the severity and extent of the observed ovarian alterations: 0 = no detectable alterations; 1 = mild alterations; 2 = moderate alterations; 3 = severe alterations. The final individual score represented an overall assessment of the severity of the alterations of the ovary, collectively considering the five histopathological parameters (follicular degeneration, cytoplasmic vacuolization, disorganization of ovarioles, pyknosis, and nuclear condensation). Histological scoring was performed blinded by an experienced invertebrate pathologist (K.P.).

### 2.5. HSP70 and HSP90 Immunohistochemistry

Due to the high biological value of honey bee queens and the experimental constraints associated with maintaining individual queens under controlled laboratory conditions, a subsample of ovaries (n = 5 per group) was selected for immunohistochemical analysis. Further, 3 μm sections were mounted on positively charged glass slides (Bio-Optica, Milan, Italy) and prepared for IHC to evaluate the expression of HSP70 and HSP90 following the protocol previously described by Power et al. [58]. The sections were deparaffinized, rehydrated in decreasing series of alcohol and subjected to antigen retrieval using citrate buffer (pH 6.0) heat treatment in a microwave oven for 6 min. Endogenous peroxidase activity was blocked prior to incubation with a protein-blocking solution for 60 min (MACH1; Biocare Medical LLC., Concord, CA, USA).

Then, the slides were incubated overnight at 4 °C with the primary antibodies anti-HSP70 and anti-HSP90 diluted in phosphate-buffered saline (PBS). The dilution used for the primary antibodies is provided in Table 1. Detection was performed using the MACH1 Universal HRP-Polymer Detection Kit (Bio-Optica, Milan, Italy). To reveal immunolabeling, 3′3-diaminobenzidine tetrahydrochloride was used as a chromogen and sections were subsequently counterstained with hematoxylin.

The immunoreactivity score (IRS) was calculated following Remmele and Stegner [59]. Staining intensity (IS) was graded as 0 (no staining), 1 (weak), 2 (moderate) and 3 (strong), while the percentage of positively stained cells (PS) was scored as 0 (<5%), 1 (5–25%), 2 (26–50%), 3 (51–75%) and 4 (>75%). The IRS was obtained by multiplying IS by PS, providing an integrated evaluation of both staining intensity and distribution.

For each queen, three histological serial sections were evaluated, and ten representative microscopic fields were examined per section at high magnification (40×). The same ovarian region was assessed across individuals to ensure consistent comparisons between control and acetamiprid-treated groups. Immunohistochemical scoring was performed blinded by an experienced invertebrate pathologist (K.P.) to minimize observer bias.

### 2.6. Western Blot

Western blot analysis was performed to verify the specificity of the immunohistochemical signals. A total of ten whole-body bee samples were lysed in ice-cold RIPA buffer supplemented with protease inhibitors, as previously described [60]. Homogenates were centrifuged at 13,000× *g* for 10 min at 4 °C and then the concentration of protein was determined using a NanoDrop 1000 spectrophotometer (ThermoFisher Scientific, Carlsbad, CA, USA). Subsequently, a total of 90 μg per sample was separated by sodium dodecyl sulfate–polyacrylamide gel electrophoresis (SDS-PAGE) and transferred to PVDF membranes. After transferring, the membranes were blocked in 5% (*w*/*v*) non-fat dry milk in Tween 0.1% Tris-buffered saline for 1.5 h at room temperature to prevent non-specific binding. The membranes were incubated overnight at 4 °C with their respective primary antibodies diluted in 2.5% (*w*/*v*) BSA in TBS-Tween. GAPDH was included as a loading control. The antibodies and their relative dilutions are reported in Table 1. After washing with Tris-buffered saline containing 0.1% Tween-20, membranes were incubated for 1.5 h at room temperature with the corresponding HRP-conjugated secondary antibody: anti-mouse IgG-HRP (1:5000; Elabscience, Houston, USA, Cat. E-AB-1001) or anti-rabbit IgG-HRP (1:5000; Elabscience, Houston, USA, Cat. E-AB-1003). Chemiluminescent signals were generated by the horseradish peroxidase (HRP)-conjugated secondary antibody following incubation with an Enhanced Chemiluminescent solution (Clarity Western ECL, 1705061, Bio-Rad, Hercules, CA, USA) for 5 min at room temperature and were detected using the XRS+ ChemiDoc Imaging System (Bio-Rad, Hercules, CA, USA). Biological triplicates were performed to ensure the reproducibility of the results.

### 2.7. Statistical Analysis

Data were analyzed using GraphPad Prism version 11.0. Given the ordinal nature of the histological scores and the small sample size, non-parametric statistics were applied, specifically, the Mann–Whitney U-test. For the immunohistochemical data, the two-tailed exact Mann–Whitney U test was used considering the small sample size (n = 5 per group) Each queen was considered as an experimental unit and the biological replicate for all statistical analyses. Sections and microscopic fields were considered subsamples, and a single score per queen was used for statistical comparisons. For histological assessment, the overall individual histological score was compared between the control (n = 10) and treated (n = 15) groups, whereas for immunohistochemical analyses, a single semi-quantitative score per queen was compared between groups (n = 5 per group).

A *p*-value < 0.01 was considered statistically significant. Results are presented as scatter dot plots, with the median indicated by a horizontal line. Statistical significance is denoted as follows: ** *p* < 0.01 and **** *p* < 0.0001.

## 3. Results

### 3.1. Histological Results

**Figure 1:**
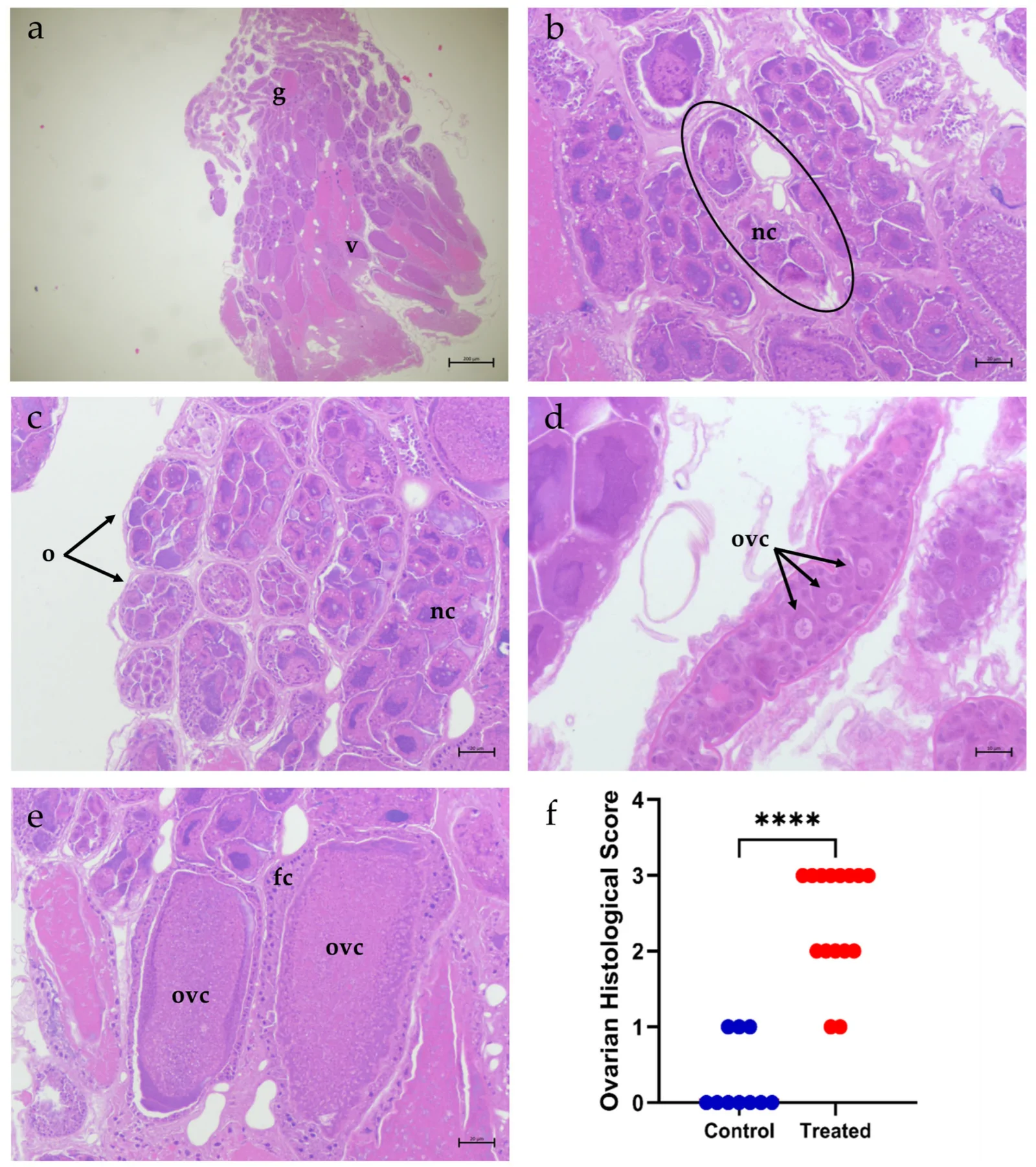
*Apis mellifera.* Normal histology of ovaries in control *Apis mellifera* queens (n = 10). (**a**) Longitudinal sections of ovarioles showing distinct apical germarium (g) and basal vitellarium (v). Note the preserved architecture and linear progression of follicles at different developmental stages. (**b**,**c**) Transverse sections illustrating multiple ovarioles (o). The nurse chambers (nc) show well-defined nurse cells with distinct nuclei; the circle in (**b**) highlights ovariole with a maturing oocyte. (**d**) Section of an ovary showing oocytes in various early stages of development. (**e**) Detailed section of the vitellarium (v) showing a maturing oocyte chamber (ovc). Note the brilliant, large nuclei (n) of the nurse cells and the continuous, well-defined follicular cell (fc) surrounding the oocyte. Staining is hematoxylin–eosin. Scale bars: (**a**) = 200 μm; (**b**,**c**,**e**) = 20 μm; (**d**) = 10 μm. (**f**) Quantitative histological assessment of ovarian tissue. Scatter dot plot showing the distribution of overall individual histological scores for control (n = 10) and treated (n = 15) groups. Horizontal lines represent the median. A highly significant structural alteration was observed in the treated group compared to controls (**** *p* < 0.0001, Mann–Whitney U test).

The ovary of the *A. mellifera* queen consists of two different areas: the germarium, which is the division zone, and the vitellarium, which corresponds to the growth zone (Figure 1a). In honey bees, oogenesis is of the meroistic polytrophic type, whereby the oocyte develops in close association with its own nurse cells (trophocytes). Histological analysis revealed a highly significant increase in the histological score of treated queens (median = 3.0) compared with control queens (median = 0.0; Mann–Whitney U-test, *p* < 0.0001; Figure 1f).

In control queens, ovarian structure displayed a well-preserved organization. The germarium exhibited a tight cellular arrangement with germinal cells presenting strongly basophilic cytoplasm and clearly visible nuclei (Figure 1a). In the vitellarium, the ovarioles were well-separated and clearly distinct (Figure 1b,c) and nurse cells were characterized by euchromatic nuclei, indicating normal cellular activity (Figure 1b). Moreover, the oocyte chambers showed spherical, homogeneous cytoplasm surrounded by continuous follicular epithelium (Figure 1d,e).

**Figure 2:**
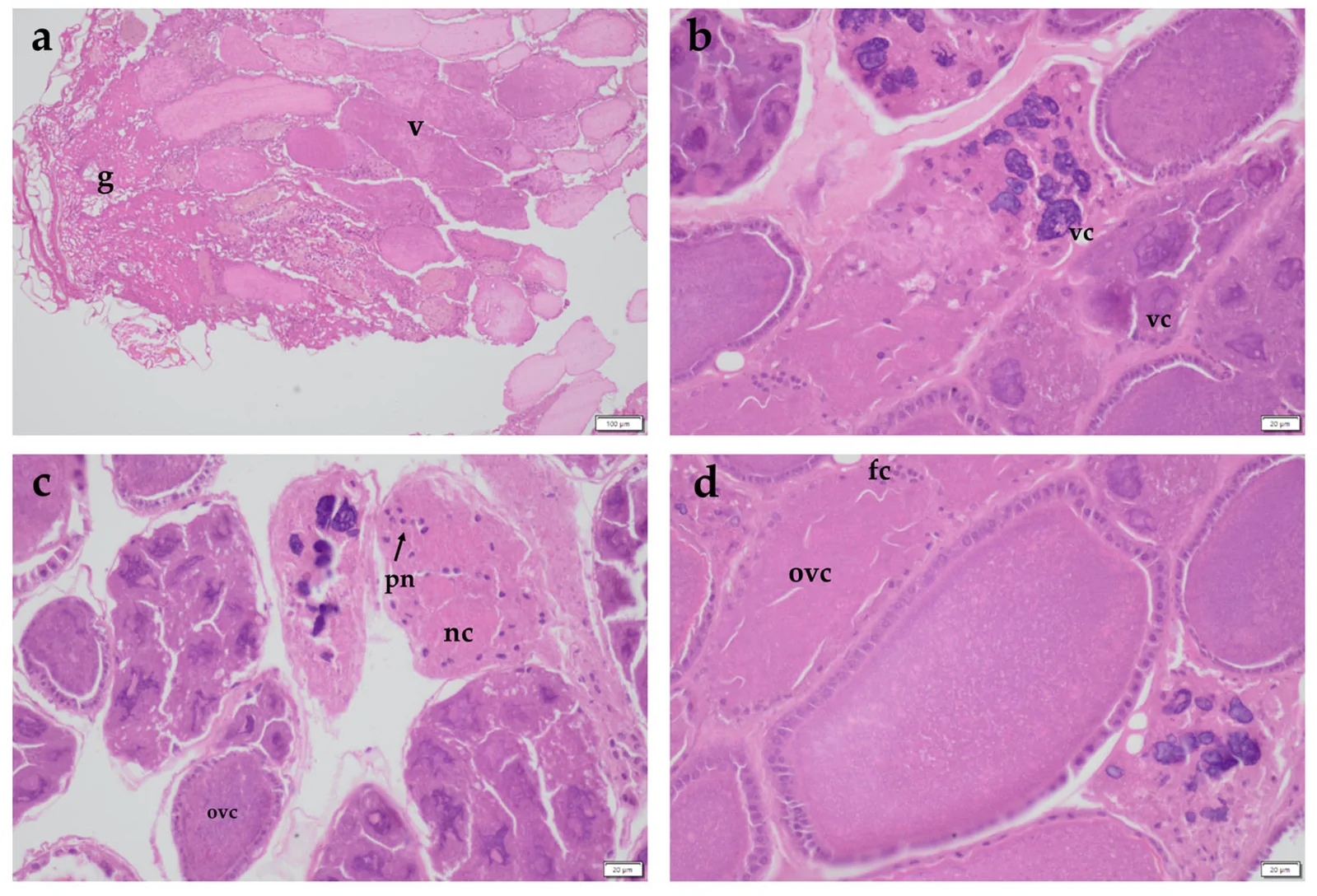
*Apis mellifera.* Histopathological alterations in the ovary of queens exposed to acetamiprid (20 µg/L) (n = 15). (**a**) Overview of an ovariole showing severe architectural disorganization and degeneration of both the germarium (g) and vitellarium (v). (**b**) Disorganization of nurse cell chambers with distinct cytoplasmic vacuolation (vc). (**c**) Detail of degenerated ovarioles. Disorganized follicular epithelium with degenerated nurse cells (nc) showing pyknotic nuclei (pn). Degenerated oocyte chambers (ovc) display fragmented cytoplasm and loss of cellular integrity. (**d**) Degenerated oocyte chamber (ovc) and disorganization of follicular epithelium (fc). Staining is hematoxylin–eosin. Scale bars: (**a**) = 100 μm; (**b**–**d**) = 20 μm.

In contrast, queens exposed to 20 μg/L of acetamiprid exhibited marked disorganization of the germarium (Figure 2a). Significant vacuolization associated with degeneration was observed in the interstitial connective tissue, resulting in reduced tissue density and architectural disorganization (Figure 2b). In the ovarian follicles, the oocytes exhibited an irregular morphology and appeared degenerated. In addition, nurse cells showed pyknotic nuclei, characterized by nuclear darkening and shrinkage (Figure 2c,d).

### 3.2. Immunohistochemical Results

**Figure 3:**
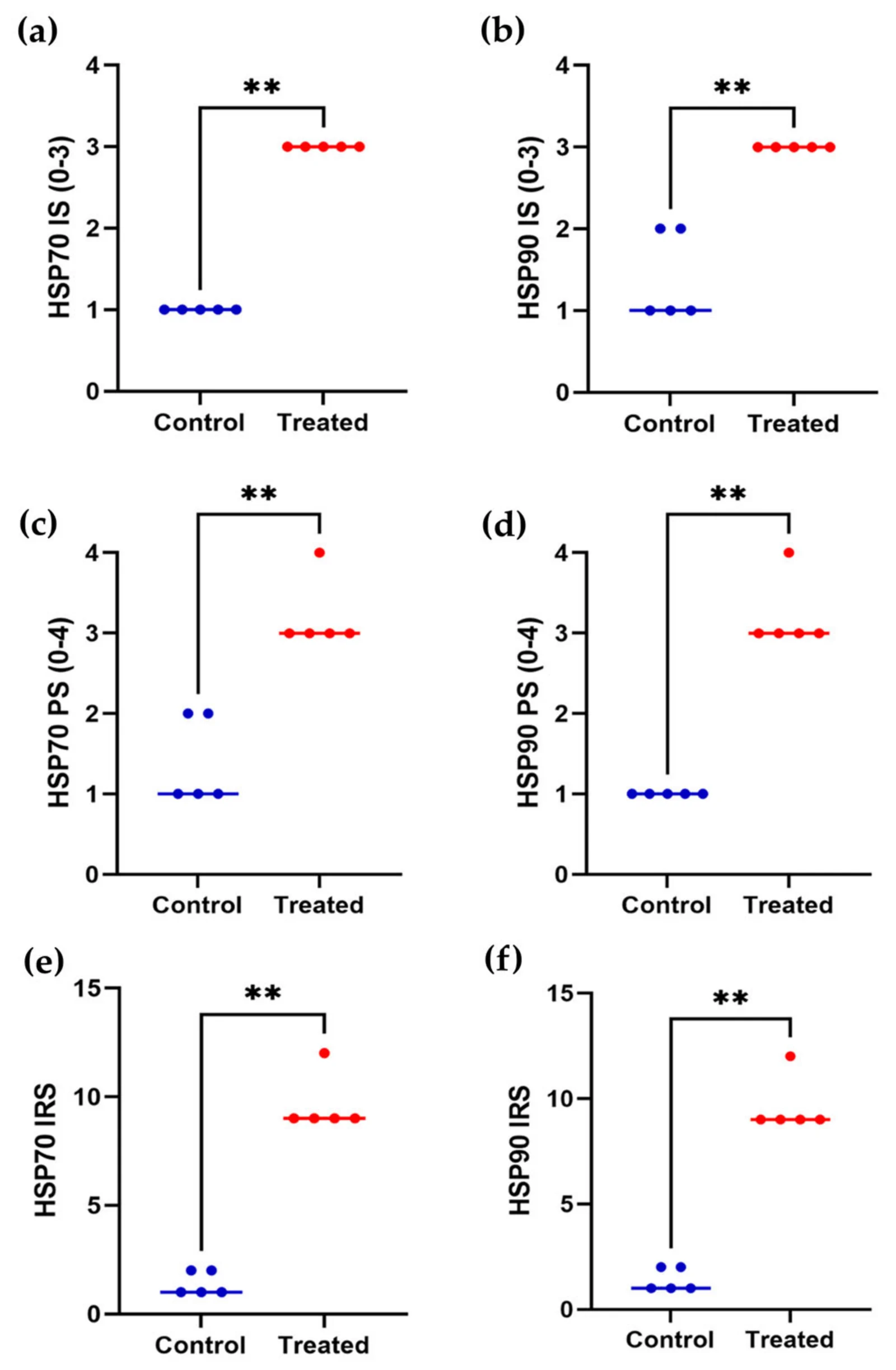
Semi–quantitative analysis of heat shock protein HSP70 and HSP90 expression in queen ovaries. Scatter dot plots showing semi–quantitative immunohistochemical scores (IS) for HSP70 (**a**) and HSP90 (**b**), the percentage of positively stained cells (PS) for HSP70 (**c**) and HSP90 (**d**), and the total immunoreactivity score (IRS) for HSP70 (**e**) and HSP90 (**f**) in control and treated groups. Horizontal lines represent the median. Statistical significance was determined using the Mann–Whitney U test (** *p* < 0.01; exact *p* value = 0.0079). Control (n = 5). Treated (n = 5).

**Figure 4:**
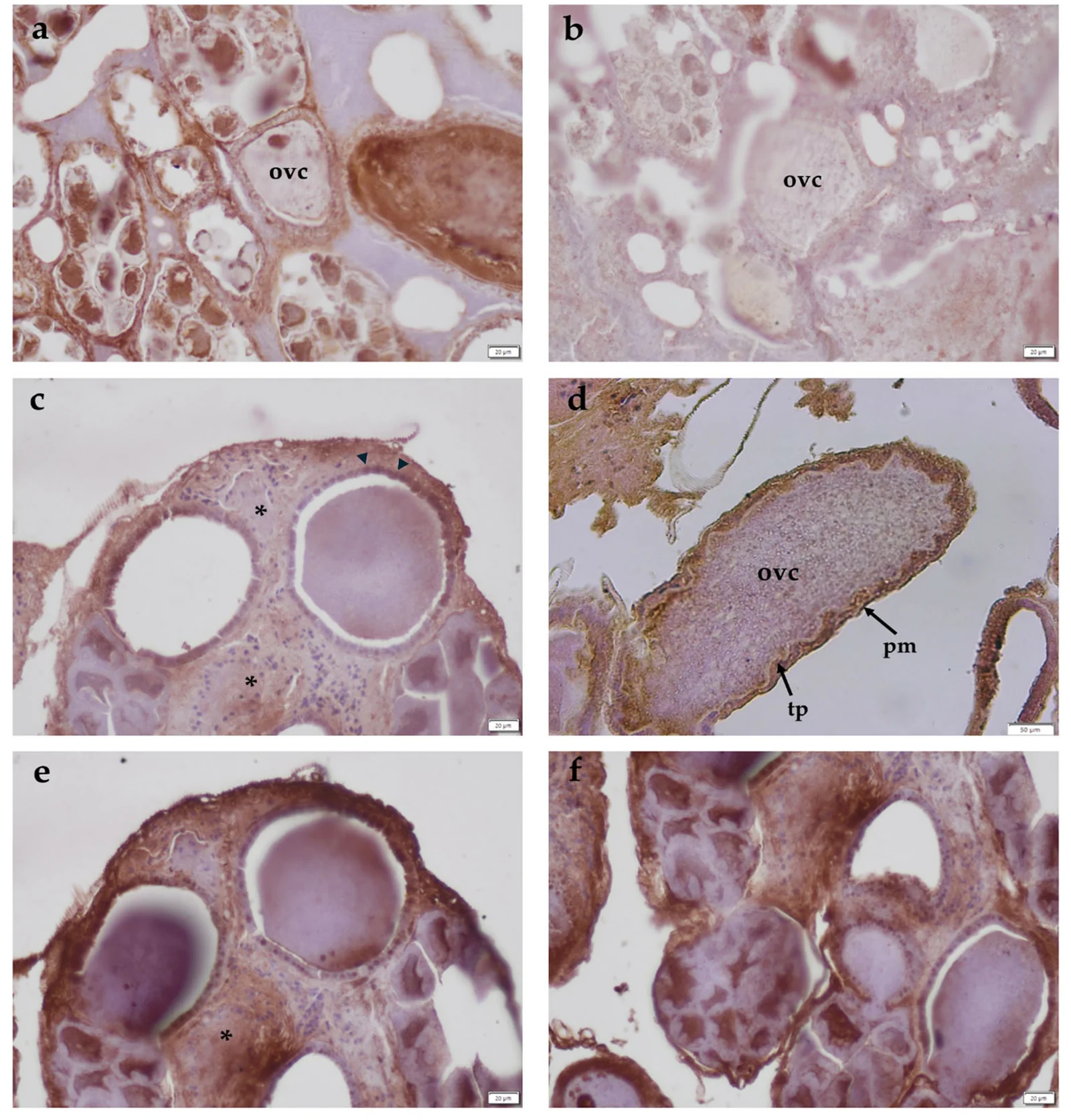
*Apis mellifera.* Immunohistochemical localization of HSP70 and HSP90 in the ovary of the *Apis mellifera* queen. Representative ovarian sections from the control group (**a**,**b**) (n = 5) and acetamiprid-treated group (**c**–**f**) (n = 5). (**a**,**b**) Control group showing weak immunoreactivity in the ovarian tissue; (**c**,**d**) HSP70 immunostaining in the acetamiprid-treated group showing increased immunoreactivity in the cytoplasm of nurse cells (asterisk) and the nuclei of follicular cells (arrow heads), in the tunica propria (tp) and peritoneal membrane (pm); (**e**,**f**) HSP90 immunostaining in the acetamiprid-treated group showing increased immunoreactivity in the cytoplasm of nurse cells (asterisk). Scale bars: (**a**–**c**,**e**,**f**) = 20 μm; (**d**) = 50 μm.

Immunohistochemical analysis revealed a significant increase in HSP70 in the treated group compared to the control group (Figure 3e). Similarly, HSP90 levels were significantly higher following acetamiprid exposure compared to controls (Figure 3f). This upregulation was evidenced by intense brown chromogenic staining (3′3-diaminobenzidine, DAB), while control samples showed only faint basal immunoreactivity for both antibodies (Figure 4a,b).

HSP70 immunoreactivity was localized in both the cytoplasm and nuclei of the nurse cells and follicular cells (Figure 4c,d), while HSP90 immunoreactivity was predominantly localized in the cytoplasm (Figure 4e,f).

### 3.3. Western Blot Results

**Figure 5:**
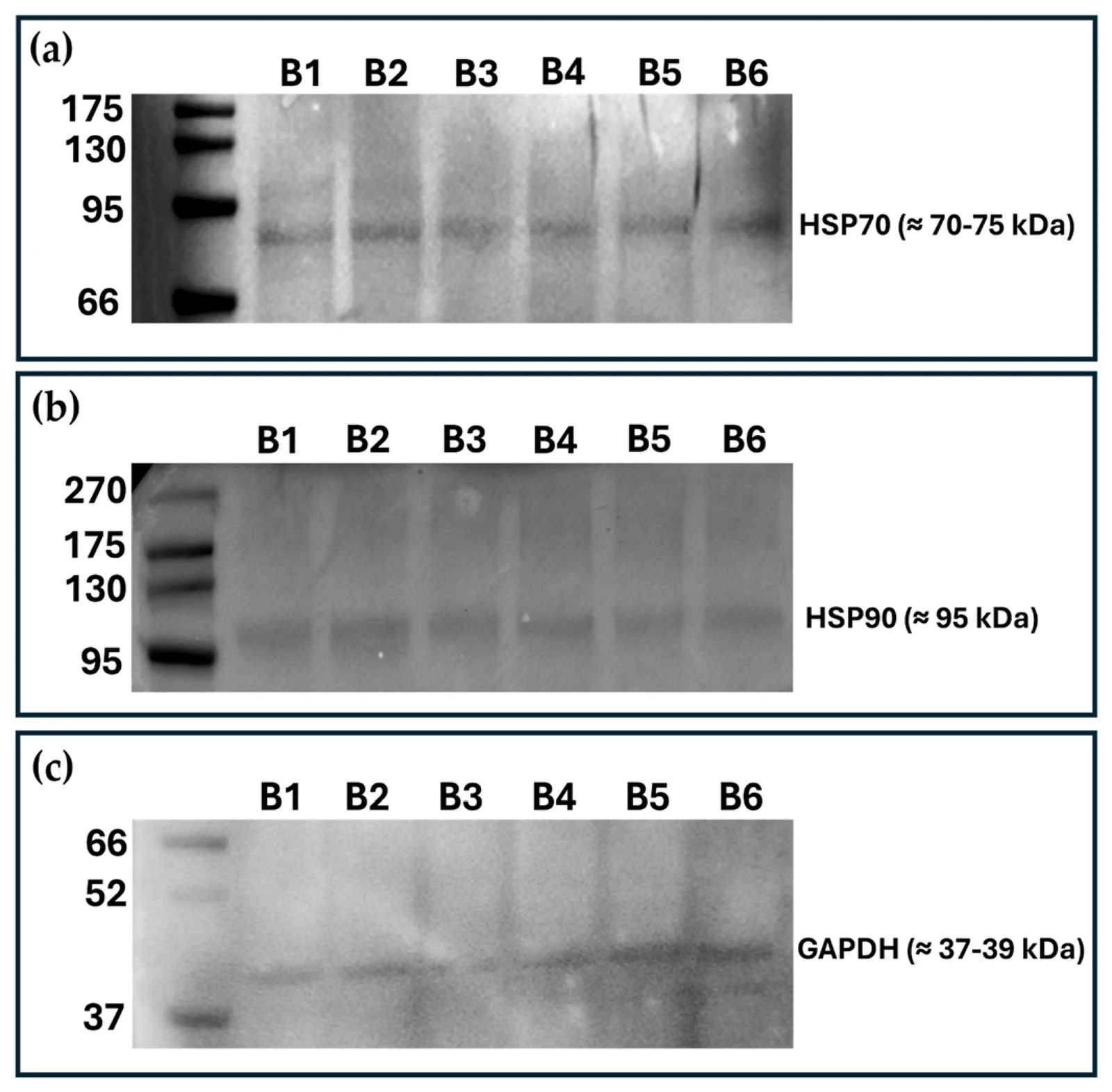
Representative HSP70, HSP90 and GAPDH immunoblot analysis. Immunoblot analysis was performed on total protein lysates obtained from bee samples, and 90 μg of protein was loaded for each lane. (**a**) HSP70 immunoblot showing a single immunoreactive band at approximately 70–75 kDa. (**b**) HSP90 immunoblot showing a single immunoreactive band at approximately 95 kDa. (**c**) The immunoreactive band against GAPDH was used as a loading control. The membranes were cut using the protein ladder as a reference. Lanes B1–B6 correspond to six representative samples (The original Western blotting images can be found in Figures S1–S3).

Western blot analysis was performed to verify the specificity of the mouse anti-HSP70 and mouse anti-HSP90 antibodies in the total protein lysates obtained from whole-body worker bee samples. As displayed in Figure 5a,b, the anti-HSP70 and anti-HSP90 antibodies detected a single immunoreactive band at approximately 70 kDa and 95 kDa, corresponding to the expected molecular weights of the respective target proteins, HSP70. GAPDH was used as a loading control and consistently detected (Figure 5c).

## 4. Discussion

The present study investigated the sublethal histological effects of the neonicotinoid acetamiprid on the ovaries of honey bee queens, and the results show severe alterations including the degeneration of connective tissue, oocytes and nurse chambers, and apoptosis. These findings are consistent with previous studies investigating the effects of different neonicotinoids on the ovarian tissue [38,39]. Moreira et al. [39] reported that chronic exposure (42 days) to 1 μg/L of imidacloprid induced changes in the morphology of both follicular and nurse cells, a decrease in the size of nurse chambers, degeneration of oocytes and resorption of ovarian content. These lesions overlap with those observed in our study already after 7 days of exposure; therefore, we could suggest that degeneration processes could start earlier than previously described. In a different study, queens exposed to 5 and 50 ng of thiamethoxam presented numerous apoptotic cells within the ovarian germarium; however, it seemed that the apoptotic incidence rate was highly variable between queens [38]. In our study, results appeared less variable between individuals, probably due to reduced genetical variability deriving from the queens being sisters. While genetic proximity reduces inter-individual variability, the generalization of our results can only be ascertained after repeating the study using queens from different genetic strains. Nevertheless, the adverse effects of acetamiprid on the queen ovary seem to overlap with those exerted by other neonicotinoids, suggesting a conserved mechanism of action on those tissues across different molecules.

Neonicotinoids are agonists of nicotinic acetylcholine receptors (nAChRs) and can induce neuronal deactivation in the mushroom bodies of honey bee brains [61,62]. Indeed, Erban et al. [52] demonstrated that one-month exposure to the same concentration of acetamiprid (20 μg/L) used in this study significantly reduced farnesyl pyrophosphate synthase (FPPS), disrupting juvenile hormone (JH) biosynthesis, which plays a crucial role in oocyte maturation and vitellogenin uptake during oogenesis, and reducing queen reproductive capacity. Although no analysis was performed on the hormonal status of queens in the present study, the hypothesis of central hormonal unbalance should be taken into consideration and further investigated. Similarly, recent studies have revealed the presence of nAChRs in tissues different from the brain [63]; however, the lack of analysis on the ovarian tissue of queens keeps still open the possibility of a direct action of neonicotinoids on the ovarian tissue.

Immunohistochemical analysis in our study revealed a marked expression of HSP70 and HSP90 within the ovarioles of exposed honey bee queens, suggesting possible disruption of protein homeostasis (proteostasis). HSPs are widely recognized as key components of the cellular stress response, where they act as molecular chaperones involved in protecting cells against damage induced by environmental stressors, including pollutants and pesticides [45]. Therefore, the overexpression of HSP70 and HSP90 likely reflects a cellular stress response to acetamiprid-induced damage. In addition, neonicotinoids are known to disrupt mitochondrial respiration, leading to increase the production of reactive oxygen species (ROS) [64]. Consequently, the upregulation of HSPs could be linked to the prevention of toxic protein aggregation induced by oxidative stress [65]. Indeed, it is well known that acetamiprid is able to affect the redox balance by reducing antioxidant activity and increasing hydrogen peroxide and malondialdehyde levels [66].

The immunolocalization findings of HSP70 and HSP90 in our study are consistent with those of Smodiš Škerl and Gregorc [43], who reported both nuclear and cytoplasmic localization of HSP70 and cytoplasmic localization of HSP90 in the hypopharyngeal glands of honey bee workers exposed to imidacloprid and coumaphos, suggesting a shared localization pattern regardless of the particular molecule. Notably, HSP70 can be physiologically expressed in the nucleus where it stimulates protein transportation and assists correct folding of newly synthetized polypeptides. In our study, the overexpression of HSP70 localized in the nuclei of nurse cells coincides with the zones of nuclear pyknosis observed in our histological sections. This nuclear translocation may indicate an adaptive response by HSP70 to protect chromatin integrity and stabilize pre-mRNAs against the genomic damage induced by toxic stress [67,68]. In contrast, the strong cytoplasmic immunoreactivity of HSP90 provides further insight into this functional decline. HSP90 is involved in an anti-apoptotic mechanism protecting cells from oxidative stress and mitochondrial disfunction, such as that caused by exposure to neonicotinoids. However, more in-depth studies using biomolecular techniques are needed to confirm those hypotheses.

Finally, it could be discussed that the degeneration of the ovaries could have derived from inadequate feeding from the attendants during the experimental time, given that acetamiprid can induce the degeneration of the hypopharyngeal glands [43]. Although reduction in egg-laying and oogenesis termination [69,70] can be associated with nutritional stress, we believe that adequate feeding of the tested individuals during the larval period and the short time of the experiment reduced the possible damage to the hypopharyngeal glands of the attendants and nutritional stress. Furthermore, control and tested individuals were maintained in the same conditions and therefore, since no alterations were observed in the controls, we can most likely attribute our results to the administration of acetamiprid.

## 5. Conclusions

Although the experiment was performed on a limited number of individuals, our results provide a first glance at the sublethal effects of acetamiprid on the ovaries of honey bee queens. The histopathological approach offers the opportunity to show microscopic changes before more classical ecotoxicological end-points are reached, and provides a chance to understand pathological mechanisms, especially when combined with immunohistochemistry and molecular biology techniques. In this study, our results suggest a possible effect of acetamiprid directly on the reproductive system and the possible protective and anti-apoptotic role of HSP70 and HSP90 on the ovarian tissue.

## Figures and Tables

**Table 1:** Primary antibodies used for immunohistochemistry and Western blot analysis.

Antibody	Target	Host Species	Clone	Manufacturer	Dilution	Catalog Number
HSP70 Monoclonal	Heat Shock Protein 70	Mouse	5C3	Elabscience, Biotechonology Inc., Houston, TX, USA	IHC:1:200; WB:1:1000	E-AB-22005
HSP90 alpha Monoclonal	Heat Shock Protein 90	Mouse	1F6	Elabscience, Biotechnology Inc., Houston, TX, USA	IHC:1:200; WB:1:1500	E-AB-22160
GAPDH Polyclonal	GAPDH	Rabbit		Elabscience, Biotechnology Inc., Houston, TX, USA	WB:1:2000	E-AB-40516

## Data Availability

The original contributions presented in this study are included in the article/Supplementary Materials. Further inquiries can be directed to the corresponding author.

## Supplementary Materials

The following supporting information can be downloaded at https://www.mdpi.com/article/10.3390/vetsci13090876/s1, Figure S1: Original images of Western blotting for HSP70; Figure S2: Original images of Western blotting for HSP90; Figure S3: Original images of Western blotting for GAPDH.
