# Supplementary material for: Sublethal Acetamiprid Exposure Induces Ovarian Degeneration and HSP70/HSP90 Overexpression in Honey Bee Queens (Apis mellifera)
Source: Vet Sci. 2026 Aug 27;13(9):876. doi: 10.3390/vetsci13090876 (PMC13611986; doi:10.3390/vetsci13090876)
Supplement: Supplementary file 1 [file vetsci-13-00876-s001.zip › vetsci-4493427-supplementary.pdf]

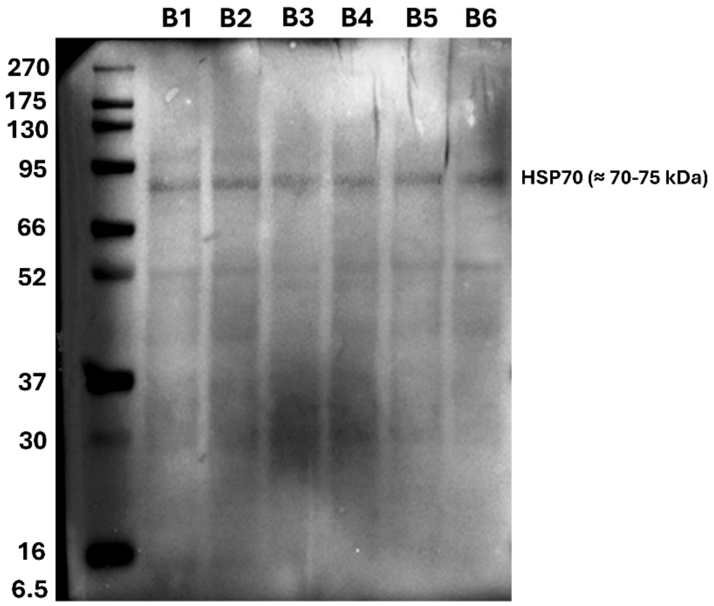

Figure S1: Original images of Western blotting for HSP70.

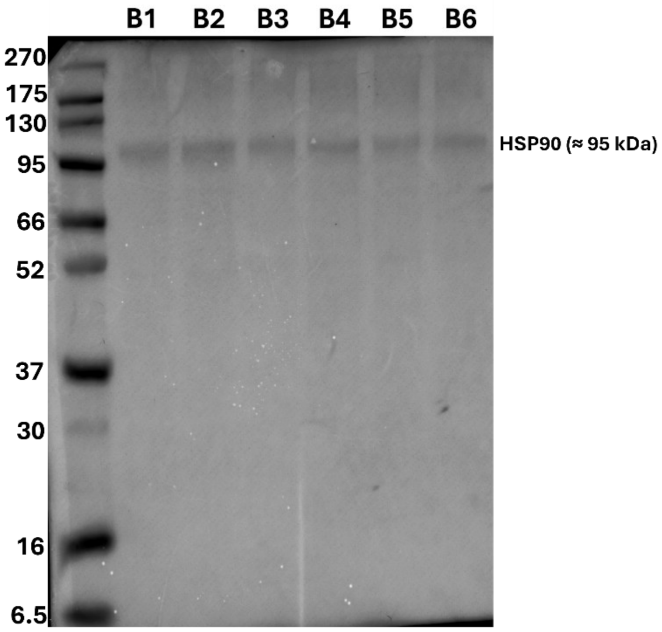

Figure S2: Original images of Western blotting for HSP90.

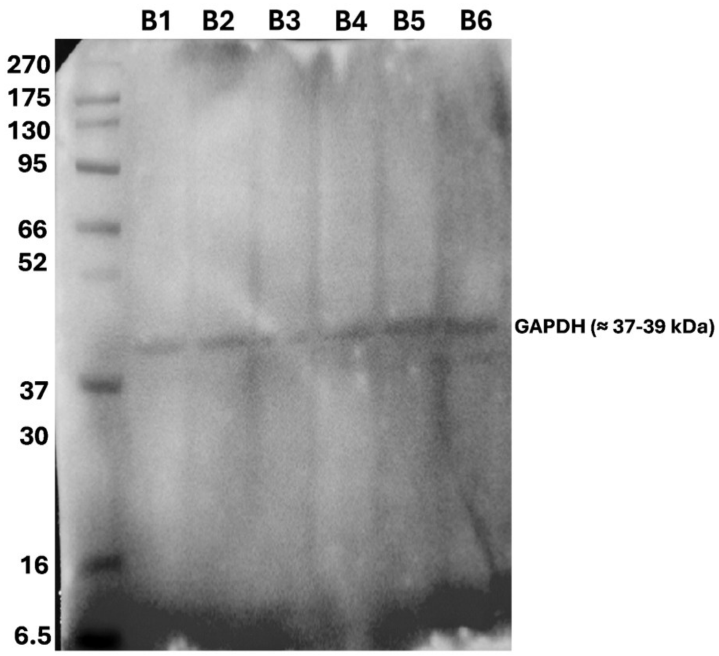

Figure S3: Original images of Western blotting for GAPDH.
